# Cochlear hair cell densities in the rabbit

**DOI:** 10.1007/s12565-018-0461-y

**Published:** 2018-10-01

**Authors:** Fang Yuan, Dalian Ding, Yitan Cao, Weidong Qi

**Affiliations:** 1 Department of Otolaryngology Head and Neck Surgery, Huashan Hospital Fudan University, 200040 Shanghai, China; 2 Department of Communicative Disorders and Sciences, Center for Hearing and Deafness, State University of New York at Buffalo, New York, Buffalo 14214 USA

**Keywords:** Cochleogram, Hair cell, Quantitative analysis, Rabbit

## Abstract

A typical cochleogram was plotted to investigate hair cell densities as a percentage along the whole length of the basilar membrane (BM) of the rabbit, the length of the BM and the width of the organ of Corti. We generated surface preparations of cochlea from adult, healthy New Zealand White (NZW) rabbits. The numbers of inner hair cells (IHCs) and outer hair cells (OHCs) were counted from images acquired from a digital camera attached to an Olympus light microscope with a scale of 100 μm as a primary unit drawn continuously, and the numbers of IHCs and OHCs were converted to densities at 10% intervals along the length of the cochlea. Meanwhile, the length of the BM and the width of the organ of Corti were calculated. The average length of the cochlea was 14.504 ± 0.403 mm, while the total number of IHCs and the numbers of OHCs in the first, second, and third rows were 1556 ± 13, 1840 ± 47, 1842 ± 46, and 1840 ± 45, respectively, accounting for 21.98%, 26.00%, 26.02%, and 26.00% of the total number of cells, respectively. The densities of each row of OHCs reported in 10% intervals were greater than the densities of the IHCs corresponding to their anatomical locations within the cochlea. The densities of OHCs in each row were distributed uniformly along the BM, while the IHCs densities were not and showed a bimodal distribution with a maximum density at the apex and at 70–80% of the cochlear length from the apex but a lower density in the remaining cochlea. The width of the organ of Corti decreased successively from the apex to the base.

## Introduction

Cochlear hair cells are the primary receptors in the inner ear that perceive sound-induced vibrations. Cochlear hair cell loss is one of the common causes of permanent sensorineural deafness and is caused by ototoxic drugs, heavy metal poisoning, high-intensity noise, pesticide poisoning, and other factors (Ding et al. 2012; Li et al. 2015; Prakash Krishnan Muthaiah et al. 2017; Yu et al. 2015). Surface preparations of the cochlea and basilar membrane (BM) must be prepared to count hair cells and generate a cochleogram to quantitatively evaluate the extent of cochlear hair cell loss in different locations along the entire length of the cochlear BM. Quantitative observations designed to evaluate the loss of cochlear hair cells have been used widely in various common experimental animal models, including guinea pigs, chinchillas, rats, and mice (Ding et al. 2011, 2013; Muller et al. 2005). However, detailed information about cochlear hair cell quantification has not yet been reported in rabbits.

Although the rabbit is a commonly used experimental animal model, rabbits are used far less frequently in auditory research than in other medical studies. In fact, the rabbit has very keen hearing and it is almost their most vital sense. In particular, the large pinna of rabbit helps them detect faint sounds over long distances. The frequency of rabbit hearing ranges from 360 to 42,000 Hz (Heffner and Masterton 1980; Martin et al. 1980), whereas the human frequency ranges from 64 to 23,000 Hz. Therefore, the hearing of rabbits is much sharper than that of humans. Rabbits have been used in ototoxicity (D’Yakonova et al. 2017), noise-induced hearing loss (Luebke et al. 2014; Moussavi-Najarkola et al. 2012), presbycusis (Bhattacharyya and Dayal 1989), and audiological (Martin et al. 2016; Peacock et al. 2015) research. In all previous publications reporting a pathological evaluation of the rabbit cochlea, researchers simply examined a small area of the cochlea because a reliable method to locate and quantify the cochlear hair cells along the entire length of the cochlear BM was not available. The purpose of this study was to provide a more detailed description of the characteristics of rabbit cochlear sensory hair cells, including the total length of the cochlear BM, the width of the organ of Corti along the cochlear BM, and the hair cell density along the cochlear BM from the apex to the basal turn.

## Materials and methods

Eight adult (2–3 months of age), healthy New Zealand White (NZW) rabbits were purchased from Slac Laboratory Animals (Shanghai, China). All experimental procedures were approved by the Institutional Animal Care and Use Committee (IACUC) of Fudan University. Animals were decapitated after intraperitoneal injections of a mixture of xylazine (10 mg/kg) and ketamine (50 mg/kg) anesthetics, and the cochleae were dissected in phosphate-buffered saline (PBS). A small hole with a diameter of 0.5 mm was drilled in the cochlea apex, the stapes were removed, and a slit in the round window was made to gently circulate 10% formalin in PBS through the apex to the basal turn with a pipette under a microscope. All specimens were immersed in 10% formalin in PBS (4 °C) overnight, rinsed with 0.1 M PBS three times and maintained in 10% EDTA in PBS for 2 weeks. Afterwards, the BM was dissected from the cochlea into segments of two-thirds of the turns and stained with TRITC-conjugated phalloidin (Sigma, P1951); finally, the segments were mounted on a slide in glycerol as a surface preparation.

**Fig. 1:**
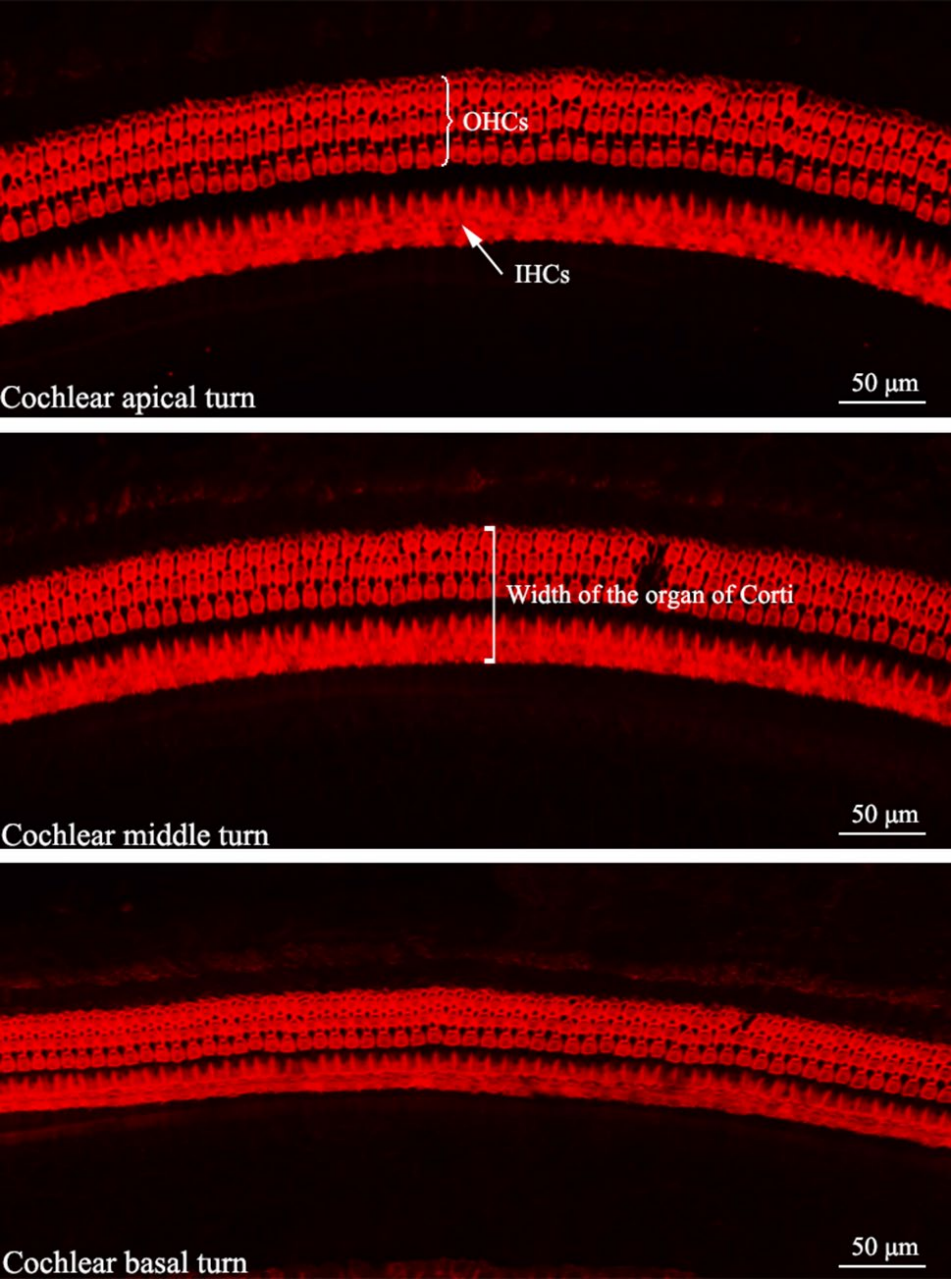
Representative images of surface preparations of the New Zealand White (NZW) rabbit cochlea (× 400, TRITC-conjugated phalloidin staining). Three rows of outer hair cells (OHCs) and the single row of inner hair cells (IHCs) are visible

Images (Fig. 1) were acquired with a digital camera attached to an Olympus light microscope (IX73). Only five well-dissected cochleae from different animals were examined. The number of inner hair cells (IHCs) and outer hair cells (OHCs) in each row on the BM were calculated from these digital images with a scale of 100 μm as a primary unit drawn continuously using the software provided with the microscope. Each 100 μm segment represents a certain percentage of the total cochlear length. The length of each BM was then acquired, and the results were averaged. We converted the length of each BM into a percentage instead of millimeters and calculated the density of IHCs and OHCs at 10% intervals along the whole length of the BM, and the results were averaged. The width of the organ of Corti was estimated from the inner margin of the IHCs to the outer margin of the OHCs at 0.5 mm intervals from the apex to the basal turn in each specimen, and then the average value was calculated for every 20% increase in length of the BM from the apex to the basal turn.

## Results

**Fig. 2:**
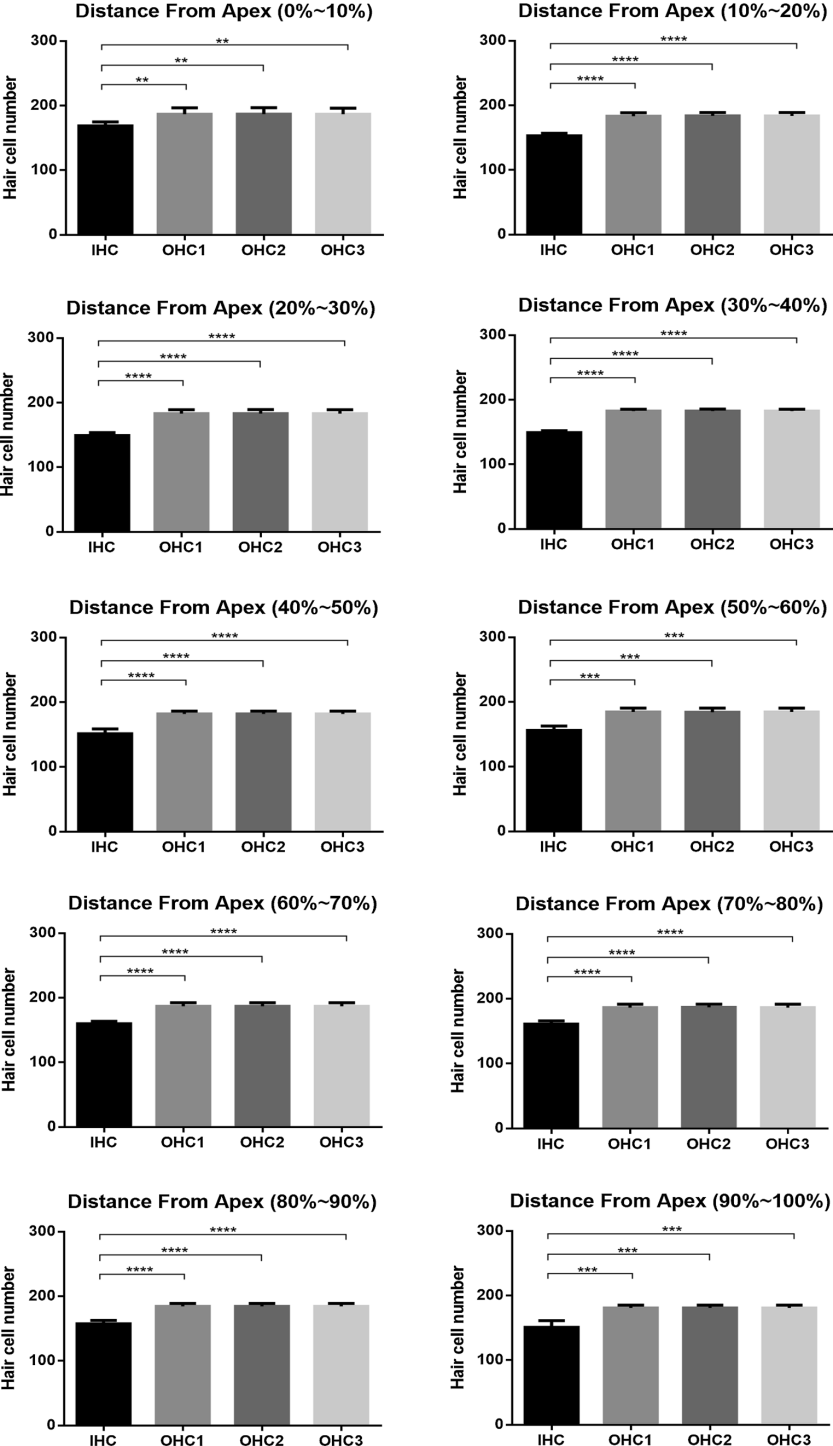
Densities of OHCs in each row reported in 10% intervals were higher than those of IHCs (*T* Test, **P < 0.01, ***P < 0.001, and ****P < 0.0001)

**Fig. 3:**
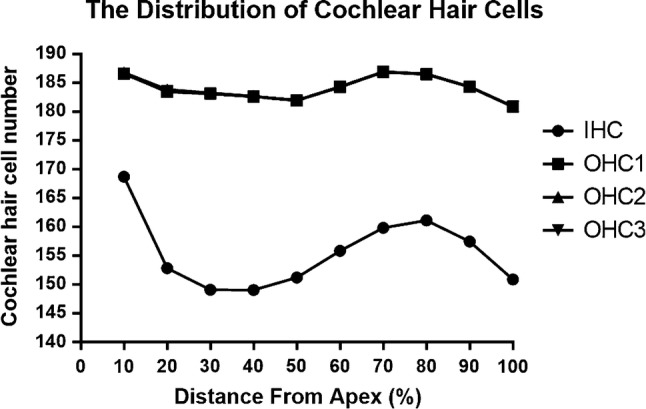
Distribution of hair cell densities along the length of the cochlea at 10% intervals. The densities of the IHCs showed a bimodal distribution, with a maximum density at the apex and at 70–80% of the cochlear length from the apex but a reduced density in the remaining cochlea

**Fig. 4:**
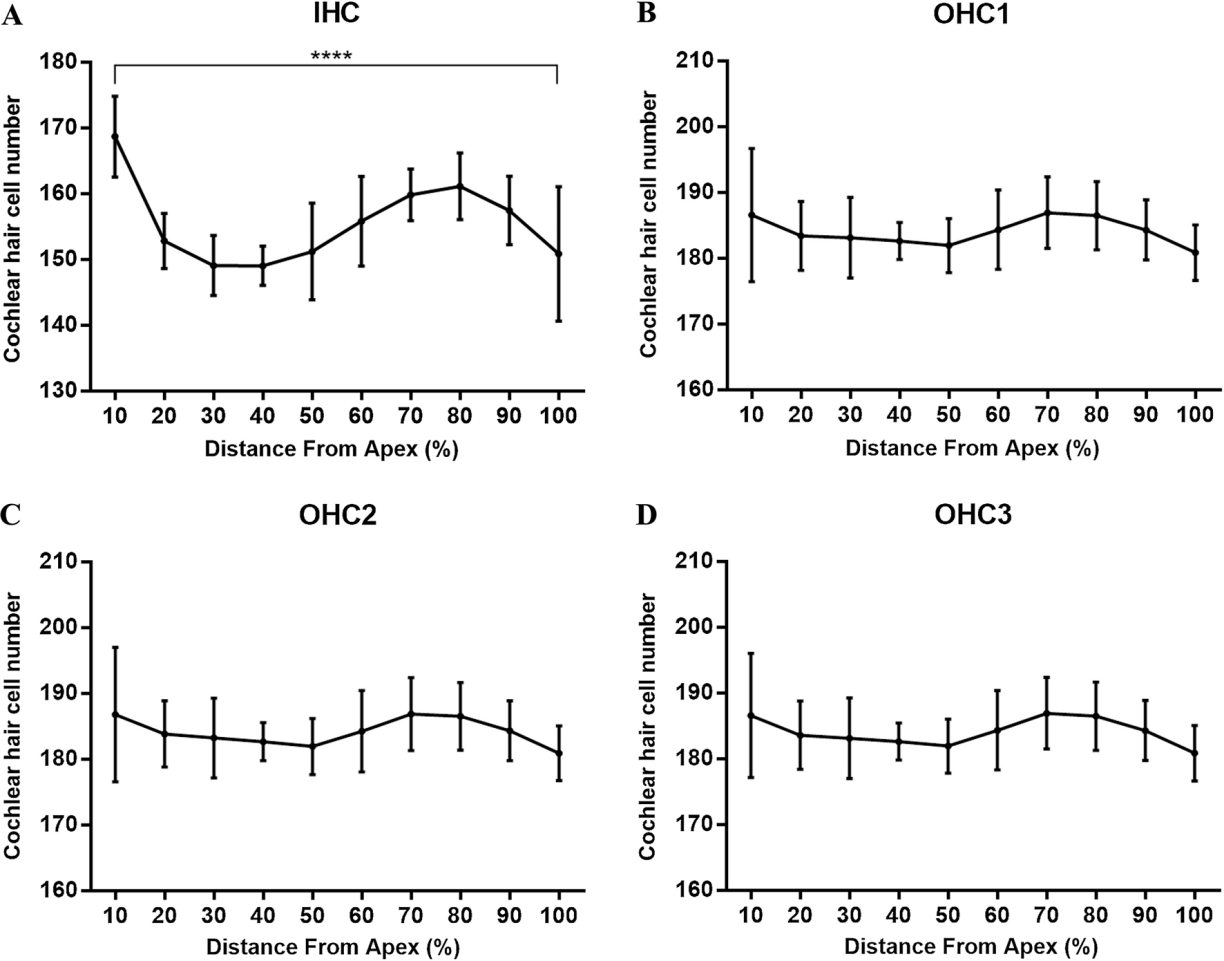
Variation in cochlear hair cell densities in the NZW rabbit along the whole length of cochlea. **a** Significant differences in IHC densities were observed along the whole length of cochlea (ANOVA, ****P < 0.0001). **b** OHC1, **c** OHC2 and **d** OHC3 densities along the whole length of cochlea were not significantly different (*P* > 0.05)

**Fig. 5:**
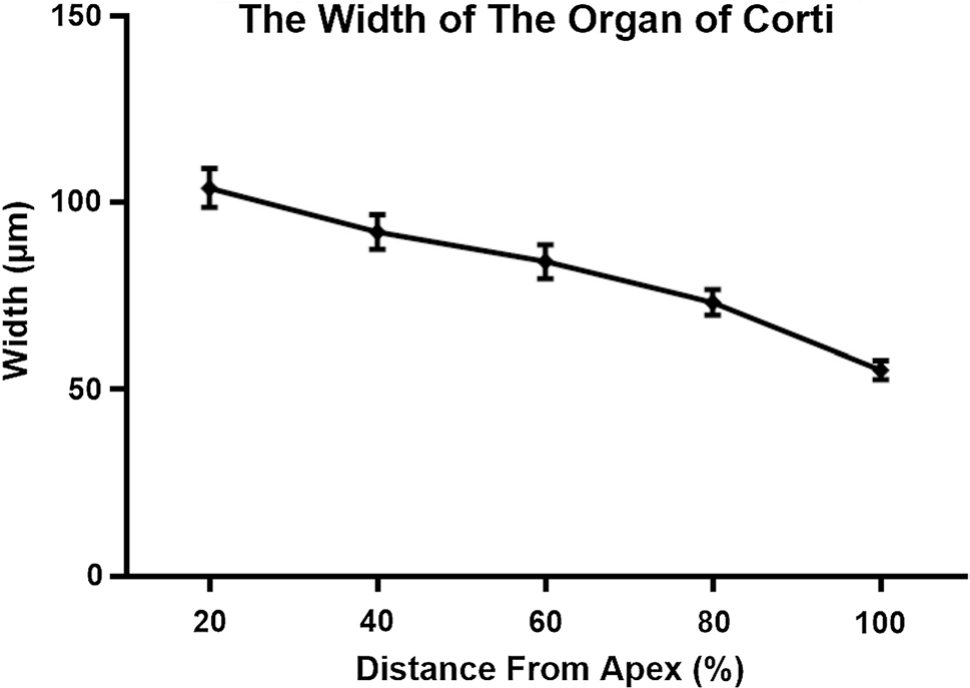
Width of the organ of Corti along the length of the cochleae at 20% intervals

For the individual analysis of each cochlea, the whole length of the BM, including the hook, was considered. Only sections of the whole cochlea with a good preparation were processed. The mean value of the cochlear length of the rabbits was calculated to be 14.504 ± 0.403 mm ($\bar{X}\; \pm \;{\text{SD}}$, *N* = 5). Hair cell densities are reported in 10% intervals in Table 1. The numbers of IHCs and OHCs in the first, second, and third rows accounted for 21.98%, 26.00%, 26.02%, and 26.00% of the total number of cells, respectively, and the results revealed higher densities of OHCs in each row in 10% intervals along the length of the cochlea than IHCs corresponding their anatomical locations within the cochlea (*P* < 0.01, Fig. 2). The distribution and variations in the densities of the IHCs and OHCs along the BM are illustrated in Figs. 3 and 4. OHCs were distributed uniformly along the BM, while the IHCs were not. Significant differences in the densities of IHCs were observed in 10% intervals along the whole length of the cochlea (*P* < 0.0001). The densities of the IHCs showed a bimodal distribution, with a maximum density at the apex and at 70–80% of the cochlear length from the apex but a reduced density in the remaining cochlea, while the densities of OHCs along the whole length of cochlea were not significantly different (*P* > 0.05). The width of the organ of Corti decreased successively from the apex to the basal turn (Fig. 5).

**Table 1:** Cochlear hair cell densities in New Zealand White (NZW) rabbits (*N* = 5) in successive 10% intervals along the length of the basilar membrane.* IHC* Inner hair cell,* OHC* outer hair cell

Distance from apex (%)	IHC	OHC1	OHC2	OHC3
10	169 ± 6	187 ± 10	187 ± 10	187 ± 9
20	153 ± 4	183 ± 5	184 ± 5	183 ± 5
30	149 ± 5	183 ± 6	183 ± 6	183 ± 6
40	149 ± 3	183 ± 3	183 ± 3	183 ± 3
50	151 ± 7	182 ± 4	182 ± 4	182 ± 4
60	156 ± 7	184 ± 6	184 ± 6	184 ± 6
70	160 ± 4	187 ± 5	187 ± 6	187 ± 5
80	161 ± 5	186 ± 5	187 ± 5	186 ± 5
90	157 ± 5	184 ± 5	184 ± 5	184 ± 5
100	151 ± 10	181 ± 4	181 ± 4	181 ± 4
Total	1556 ± 13	1840 ± 47	1842 ± 46	1840 ± 45
	(21.98%)	(26.00%)	(26.02%)	(26.00%)

## Discussion

Hearing loss, which is always accompanied by auditory hair cell damage, is currently evaluated with audiograms, auditory brainstem responses, otoacoustic emissions, and word recognition scores in clinical examinations. However, none of these methods replace histopathological observations of the cochlea in hearing research. Hair cells, including OHCs and IHCs in the BM, are the primary units responsible for the sense of hearing. In previous studies, distortion-product otoacoustic emissions (DPOAEs), a common method used to evaluate lesions in rabbits and provide an objective, noninvasive, and quantitative measure of OHC activity, failed to consider IHCs (D’Yakonova et al. 2017; Moussavi-Najarkola et al. 2012; Ohlms et al. 1991).

More than 50 years ago, the cochleogram was introduced as a tool to visualize properties of the cochlea in relation to the location within the cochlea using a surface preparation technique, and it represents a feasible method for assessing the damage to both the IHCs and OHCs by depicting the percentage of hair cell loss along the length of the cochlea (Saunders 1967). Since then, the cochleogram has become a routine procedure for plotting hair cell loss and correlating this parameter with physiological changes occurring in response to morphological insults. Martin applied a cochleogram to depict the percentages of missing IHCs and OHCs based on the percentage distance from the cochlear apex to evaluate the surgical destruction of the endolymphatic sac and the distal portion of the duct efficiency in a rabbit model of endolymphatic hydrops (Martin et al. 1983). Bhattacharyya depicted inner and total outer hair cell loss in rabbits at four different ages using a cochleogram (Bhattacharyya and Dayal 1989), but the cochleograms were plotted based on the actual length from the apex.

A standard cochleogram should be plotted using the following guidelines: (1) BM length should be plotted as a percentage instead of in millimeters due to the biological variation in BM length within a particular species and strain, and the total length in millimeters should be stated on the cochleogram; and (2) different BM lengths should be normalized to percentages before average cochleograms are constructed (Viberg and Canlon 2004). Therefore, we must obtain a basic understanding of some of the fundamental anatomical features of the rabbit cochlea, such as the number of hair cells, hair cell density and cochlear length.

To the best of our knowledge, systematic studies have never been performed to evaluate hair cell densities in the rabbit. Although Ramprashad (1984) had described the morphometric parameters of the rabbit cochlea, including the morphometric changes along the length of the cochlea of the cross-sectional areas of the scalae vestibuli, scalae tympani, scalae media, and BM, as well as the width and thickness of the BM and the density of the bipolar ganglion cells innervating the organ of Corti, the authors did not report hair cell densities, which is crucial for hearing research.

Since considerable intra-species variations exist in BM length and diverse methods are used for specimen fixation and measurements in research, some variations in BM length have been reported, and hence the BM length should be plotted as a percentage, and not in millimeters, on the cochleogram (Viberg and Canlon 2004); additionally, general parameters such as the complete length of the cochlea or the average hair cell density should be studied. As shown in Table 1, in which the number of hair cells is presented at 10% intervals, the densities of hair cells from different regions along the cochlea exhibit certain differences. Figures 3 and 4 illustrate the distribution and densities of OHCs in each row along the whole length of the cochlea, which present a similar slight variation, ranging in number from 181 to 187, but the differences were not statistically significant (*P* > 0.05). Compared with the OHCs, the densities of the IHCs showed a bimodal distribution, ranging in number from 149 to 169, and the differences were statistically significant (*P* < 0.0001). The densities of OHCs in each row reported in 10% intervals were higher than the IHCs corresponding their anatomical locations within the cochlea.

## Conclusions

The densities of OHCs in each row reported at 10% intervals were higher than the IHCs corresponding their anatomical locations within the cochlea (*P* < 0.01). OHCs in each row along the BM were uniformly distributed, while the IHCs were not. Significant differences in the densities of IHCs were observed in 10% intervals along the whole length of cochlea (*P* < 0.0001). The densities of the IHCs showed a bimodal distribution, with a maximum density at the apex and at 70–80% of the cochlear length from the apex but with a lower density in the remaining cochlea, while the densities of OHCs along the whole length of cochlea were not significantly different. The width of the organ of Corti decreased successively from the apex to the basal turn.

Information on the hair cell densities in the rabbit cochlea is very rare in the literature. The data acquired from this observational study provide a reference for the averaged value of hair cell densities in percentages, as well as the length of the cochlea in the NZW rabbit, which will facilitate direct comparisons when researchers plot cochleograms.
